# Anti-dsDNA antibodies bind to TLR4 and activate NLRP3 inflammasome in lupus monocytes/macrophages

**DOI:** 10.1186/s12967-016-0911-z

**Published:** 2016-06-01

**Authors:** Hui Zhang, Rong Fu, Chaohuan Guo, Yuefang Huang, Hongyue Wang, Shuang Wang, Jijun Zhao, Niansheng Yang

**Affiliations:** Department of Rheumatology, First Affiliated Hospital, Sun Yat-sen University, 58 Zhongshan Road II, Guangzhou, 510080 China; Department of Pediatrics, First Affiliated Hospital, Sun Yat-sen University, Zhongshan Road II, Guangzhou, 510080 China

**Keywords:** SLE, Anti-dsDNA antibodies, NLRP3 inflammasome, TLR4, Mitochondrial ROS

## Abstract

**Background:**

NLRP3 inflammasome has been implicated in the pathogenesis of systemic lupus erythematosus (SLE). The activation of NLRP3 inflammasome results in the production of IL-1β and the subsequent inflammation. Anti-dsDNA antibodies (anti-dsDNA Abs) play critical roles in the development and progression of SLE. However, the mechanism of NLRP3 inflammasome activation in SLE is still not known. This study investigated the activation of NLRP3 inflammasome stimulated by anti-dsDNA Abs in monocytes/macrophages from SLE patients.

**Methods:**

Monocytes/macrophages from SLE patients or healthy controls were stimulated with anti-dsDNA Ab-positive serum or purified anti-dsDNA Abs. Activation of inflammasome was measured by flow cytometry or Western blot. Anti-dsDNA Abs isolated from active SLE patients were injected into female (NZB × NZW) F1 mice and the activation of NLRP3 inflammasome and the frequencies of Th17 and Treg were examined.

**Results:**

The activity of caspase-1 was significantly increased in active SLE patients and was correlated with serum levels of anti-dsDNA Abs and disease activities. The concentrations of IL-1β and IL-17A were also significantly higher in SLE patients compared to healthy controls. Anti-dsDNA Ab-positive serum rather than healthy serum or RF (rheumatoid factor)-positive serum stimulated the activation of caspase-1 in monocytes. Anti-dsDNA Abs bound to TLR4 on macrophages and induced the production of ROS. Mitochondria-targeting antioxidant Mito-TEMPO, IκB kinase inhibitor peptide or TLR4 siRNA inhibited the activation of NLRP3 inflammasome and the secretion of IL-1β induced by anti-dsDNA Abs. Injection of anti-dsDNA Abs into (NZB × NZW) F1 mice resulted in increased caspase-1 activation and production of IL-1β and IL-17A. The Th17/Treg cell ratio also significantly increased following anti-dsDNA Ab injection.

**Conclusions:**

Anti-dsDNA Abs activated NLRP3 inflammasome in monocytes/macrophages from SLE patients by binding to TLR4 and inducing the production of mitochondrial ROS.

## Background

Systemic lupus erythematosus (SLE) is a systemic autoimmune disease with multiple organs involvement [1]. Despite the advances in the treatment of SLE, the morbidity and mortality of the disease remain high [2]. Dysregulation of immune system plays a critical role in the initiation and progression of SLE. Overproduction of cytokines and the subsequent inflammation are involved in the pathogenesis of SLE [3]. IL-1β is a proinflammatory cytokine that has been linked to SLE. It has been demonstrated that IL-1β is elevated in the serum of SLE patients [3, 4] and IL-1 receptor antagonist led to clinical and serological improvement [5]. In addition, IL-1β^−/−^ and IL-1α/β^−/−^ mice are resistant to the induction of experimental lupus [6].

Inflammasomes are a family of molecular platforms mostly expressed in the cytoplasm of monocytes/macrophages. Inflammasomes are activated by infection or danger signals and trigger the maturation of proinflammatory cytokines such as IL-1β to engage in innate immune response [7]. NLRP3 inflammasome is one of the most studied members. Activation of NLRP3 inflammasome involves the recruitment of adapter protein apoptosis-associated speck like protein (ASC) through homotypic PYD-PYD interaction. ASC subsequently recruits pro-caspase-1 via CARD-CARD contact in turn. The oligomerization of NLRP3 inflammasome leads to autocatalytic activation of caspase-1, which converts inactive pro-IL-1 into bioactive form [8]. NLRP3 inflammasome can be activated by either exogenous or endogenous stimuli [9–11].

Anti-double stranded DNA (anti-dsDNA) antibodies are the hallmark antibodies of SLE [12]. Anti-dsDNA antibodies are correlated with disease activity [13] and involved in the pathogenesis of SLE [14]. Anti-dsDNA antibodies presented in the blood before disease onset [15] and administration of anti-dsDNA antibodies from SLE patients into NZBWF1/J mice resulted in accelerated lupus [16]. Data showed that anti-dsDNA antibodies stimulated the expression and secretion of IL-1β from mononuclear cells and monocytes [17, 18]. It implies that anti-dsDNA antibodies might initiate and promote the disease by stimulating the production of IL-1β.

Recently, we have showed that NLRP3 inflammasome was activated in a mouse model of SLE, and inhibition of NLRP3 inflammsome activation resulted in decreased inflammation and improved disease severities [19, 20]. However, factors that trigger the activation of NLRP3 inflammasome in SLE patients are not well defined. In the present study, by using purified anti-dsDNA antibodies from active SLE patients, we showed that anti-dsDNA antibodies activated NLRP3 inflammasome in monocytes/macrophages by binding to TLR4 and inducing the production of mitochondrial ROS (reactive oxygen species).

## Methods

### Patients

In this study, 72 patients from the First Affiliated Hospital, Sun Yat-sen University who fulfilled the American College of Rheumatology criteria for the classification of SLE [21] and 36 age and sex matched healthy donors were enrolled. Patients with comorbidities of cancers or infections were excluded. Disease activity was scored using SLE Disease Activity Index (SLEDAI) scoring system [22]. Only patients with moderate to severe disease activity were included in the study (defined as SLEDAI > 4) [23]. Demographic and clinical characteristics of the SLE patients are shown in Table 1.

**Table 1:** Demographical and clinical characteristics of the SLE patients (n = 72)

Age (years)	30.2 ± 12.6
Sex (male/female)	7/65
Newly-onset (no.)	27
Kidney involvement (no.)	42
ESR (mm/h)	39.1 ± 24.8
CRP (mg/L)	7.7 ± 12.3
Anti-dsDNA Abs (IU/ml)^a^	2.7 ± 1.6
ANA (IU/ml)^b^	7.4 ± 3.2
C3 (g/L)	0.5 ± 0.3
C4 (g/L)	0.1 ± 0.06
SLEDAI	9.2 ± 5.9

The healthy control group consisted 4 men and 32 women with a mean ± SD, age of 28 ± 10.7 years

*SLE* systemic lupus erythematosus, *Anti*-*dsDNA* anti-double stranded DNA antibodies, *SLEDAI* systemic lupus erythematosus disease activity index

^a,b^Concentration of 0–0.9 IU/ml was recognized as negative in this assay

### Monocyte isolation and macrophage differentiation

Human peripheral blood mononuclear cells (PBMCs) were isolated from SLE patients or from healthy controls with Ficoll-Hypaque by density gradient centrifugation. Cells were cultured in antibiotic-free RPMI 1640 medium containing 10 % FCS, 2 mM glutamate, 50 mM 2-ME, and 10 mM HEPES buffer in 24-well plates. To obtain monocytes, PBMCs were seeded to the culture dishes for 3 h to let the monocytes to adhere. Three hours later, non-adherent cells were washed away with PBS. To obtain macrophages, isolated monocytes were cultured with culture medium with 10 ng/ml of M-CSF for 7 days as previously described [24]. Culture medium was changed every 2 days.

### Anti-dsDNA antibody isolation

Anti-dsDNA antibody-positive sera were collected from active SLE patients. Healthy sera were used as controls. Polyclonal anti-dsDNA antibodies were isolated from sera of SLE patients and control IgG from healthy subjects by affinity chromatography as we previously described [25]. Immune complexes were precipitated with polyethylene glycol (3.5 % [wt/vol]). Serum samples were then diluted with PBS and added to native DNA-cellulose column (GE Biotech, USA). Non-DNA-binding fractions were flushed. DNA binding fractions were eluted with a linear NaCl gradient. IgG was isolated with protein G Sepharose affinity chromatography kits (GE Biotech, USA) and the purities of eluted IgG were confirmed by 10 % sodium dodecyl sulfate–polyacrylamide gel electrophoresis (≥90 %).

### Cell culture

PBMCs from SLE patients or healthy controls were isolated and 1 × 10^6^ cells were seeded to 48-well culture plate. Non-adherent cells were washed away and cells were maintained at 37 °C in 5 % CO_2_ in a humidified cell culture incubator. Monocytes isolated from PBMCs were stimulated with healthy serum, rheumatoid factor (RF)-positive serum from rheumatoid arthritis (RA) patients, anti-dsDNA antibody-positive serum from SLE patients, or purified anti-dsDNA antibodies for 16 h. In addition, monocytes from SLE patients or healthy controls were stimulated with anti-dsDNA antibodies or control IgG for 16 h. Macrophages differentiated from SLE monocytes were stimulated with anti-dsDNA antibodies or control IgG for 16 h. Unless otherwise stated, drug concentrations were used as the following: Mito-TEMPO ((2-(2,2,6,6-tetramethylpiperidin-1-oxyl-4-ylamino)-2-oxoethyl) triphenylphosphonium chloride) (100 μM) (Sigma, Shanghai, China), glyburide (20 μM) (Sigma, Shanghai, China), Ac-YVAD-CMK (50 μM) (Calbiochem, Hong Kong, China) or IκB kinase inhibitor peptide (200 μM) (Calbiochem, Hong Kong, China). Cells were stimulated with 100 ng/ml of LPS for 6 h and 5 μM of ATP were included for the last 2 h, which was used as positive control. Supernatant was collected and frozen at −80 °C until tested.

### Detection of anti-dsDNA antibodies binding to TLR4

Monocytes/macrophages from SLE patients were acquired as described above. Cells were collected and incubated with anti-CD16/CD32/CD64 antibodies (Biolegend, San Diego, CA, USA) to block Fc receptor before incubated with control IgG or anti-dsDNA antibodies. Briefly, cells were incubated with control IgG (5 μg/ml) or different concentrations of anti-dsDNA antibodies at 4 °C for 1 h. Cells were washed and stained with APC-anti-Toll-like receptor-4 (TLR4) antibody (Biolegend, San Diego, CA, USA) at 4 °C for 30 min. Data were acquired by flow cytometer FACS Aria (BD Bioscience, San Jose, CA, USA).

### Transfection

One million THP-1 cells (a human monocytic cell line, ATCC, USA) were seeded to 6-well culture plates. Cells were stimulated with 100 nM of PMA for 18 h. Briefly, cells were washed and transfected with 50 nM TLR4 siRNA (Ruibo-bio, Guangzhou, China) together with Lipofectamine 2000 (Invitrogen, New York, USA) in 500 μl Opti-MEM I Reduced-Serum Medium (Invitrogen, New York, USA) at 37 °C in a CO_2_ incubator. Cells were also transfected with 50 nM of scramble siRNA complexed with Lipofectamine 2000. The RNAi-Lipofectamine complex was removed after incubated for 6 h, and the cells were cultured overnight in RPMI plus 10 % FBS. Twenty-four hours after transfection, cells were maintained with serum-free medium for 24 h prior to use. Then THP-1 cells were incubated with 10 μg/ml of anti-dsDNA antibodies or IgG control for 16 h.

### Mice experiments

Female (NZB × NZW) F1 mice (The Jackson Laboratory, USA) of 24-week of age were used in this study. Mice were kept in specific pathogen free condition in Sun Yat-sen University Animal Facility. Chow and water were supplied *ad libitum*. Mice were divided into three groups, control group, control IgG group and anti-dsDNA antibody group (n = 8). Mice received intraperitoneal injection of control vehicle, 100 μg of control IgG or 100 μg of anti-dsDNA antibodies in 100 μl PBS. The injection was repeated once 3 days later. Mice were sacrificed by cardiac puncture under anesthesia on day 7. Anticoagulated blood was collected and plasma was isolated by centrifugation, which were frozen at −80 °C until being analyzed for the concentrations of IL-1β and IL-17A. White blood cells were collected by lysing red blood cells. Spleens were collected and single cell suspension was prepared for the measurement of Th17 cells and regulatory T cells (Treg) by flow cytometry.

### Flow cytometry

To determine the activation of caspase-1 intracellularly, a fluorescence-labeled inhibitor specific for caspase-1 (FLICA) probe (ImmunoChemistry Technologies, Bloomington, MN, USA) was used as previously described [26]. Cells were collected and incubated with FLICA in 37 °C for 30 min. Cells were washed and analyzed by flow cytometry. For measuring mitochondria membrane potential (ΔΨm), Rhodamine 123 (Sigma, Shanghai, China) was used as previously described [27]. 2,7-dihydrochlorofluorescein (DHCF) (Sigma, Shanghai, Chiba) was used to measure the production of ROS in the cells [28]. To detect the frequency of Th17 cells in the spleen, single cell suspension was prepared from the spleen and 1 × 10^6^ cells was stimulated with 50 ng/ml phorbolmyristate acetate (PMA) plus 500 ng/ml ionomycin (Sigma, Shanghai, China) for 5 h, with inclusion of 10 μg/ml of brebeldin A (eBiosciences, San Diego, CA, USA) in the last 2 h. Cells were fixed, permeabilized, and stained with PE-anti-IL-17A (BD Pharmingen, San Jose, CA, USA) after stained with FITC-anti-CD4 antibodies (BD Pharmingen, San Jose, CA, USA). To analyze the frequency of Foxp3^+^ Treg in the spleen, 1 × 10^6^ cells were stained with anti-CD4 antibody (eBioscience, San Diego, CA, USA). Cells were fixed, permeabilized, and stained with PE-Cy5-anti-Foxp3 antibody (eBioscience, San Diego, CA, USA). Data was analyzed by Flowjo software (Tree Star Inc, Ashland, OR, USA).

### Western blot

Cells were collected and protein was extracted. Briefly, extracted cellular proteins were loaded to SDS–polyacrylamide gels. Protein was electrotransferred onto polyvinylidinedifluoride membranes. The membranes were blocked with 5 % bovine serum albumin in TBST and incubated with anti-ASC (Novus, Littleton, CO, USA), anti-caspase-1 p10, anti-P2X7 receptor (P2X7R), anti-Cathepsin-B (Abcam, Hong Kong, China), anti-pro-IL-1 (Santa, Cruz Biotechnology, Dallas, TX, USA), or anti-GAPDH (Kangcheng, Shanghai, China) primary antibodies at 4 °C overnight. The membranes were then washed and incubated with horseradish peroxidase conjugated anti-rabbit IgG (Cell Signaling Technology, Beverly, MA, USA) at room temperature for 60 min, and the signals were detected by enhanced chemiluminescence.

### Luminex assay

Human serum collected from SLE patients or healthy controls were frozen at −80 °C until analyzed by Luminex assay (Invitrogen, New York, USA), as measured on a Bio-Plex system (Bio-Rad, Hercules, USA). IL-1β and IL-17A were included in the multiplex analysis kit. For the measurement of IL-1β and IL-17A in mouse serum, commercial ELISA kits (eBioscience, San Diego, USA) were used according to the manufacturer’s instructions.

### Statistical analyses

The data was expressed as the mean ± SD. Statistical analysis was performed using SPSS 13.0 (SPSS Inc, Chicago, USA). The differences were assessed by *t* test, or one way ANOVA with or without repeated measurements followed by Bonferroni’s multiple comparison post test as appropriate. Correlation analyses were done by Spearman’s rank correlation test. Two-tailed *p* <0.05 was considered statistically significant.

## Results

### Inflammasome was activated in SLE and correlated with serum anti-dsDNA antibody level

**Fig. 1:**
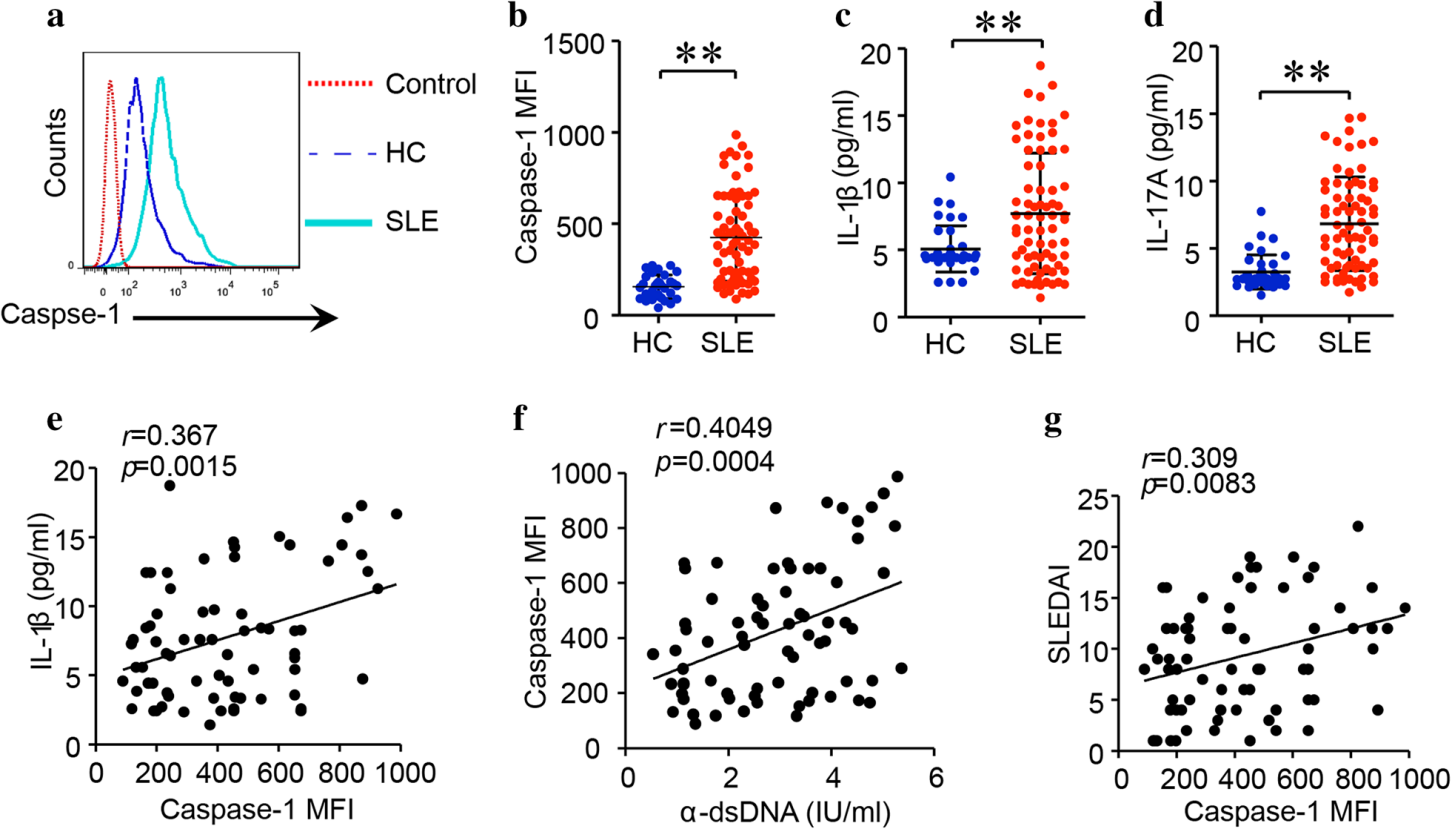
Activation of inflammasome in monocytes from active SLE patients. **a**, **b** PBMCs were isolated from active SLE patients (n = 72) or healthy controls (HC) (n = 36). The activation of caspase-1 in monocytes was measured by flow cytometry and cells were gated on monocyte population. The activation of caspase-1 in SLE patients was significantly higher than that of healthy controls. **c**, **d** Serum levels of IL-1β and IL-17A were significantly higher in active SLE patients than that of healthy controls. **e** Serum level of IL-1β was correlated with MFI of active caspase-1. **f** MFI of active caspase-1 was correlated with serum level of anti-dsDNA antibody. **g** MFI of active caspase-1 was correlated with SLEDAI. **p < 0.001, vs HC. *α*-*dsDNA* anti-dsDNA antibodies

Our previous study has shown that caspase-1 was activated in a mouse model of SLE [8]. To measure the activity of caspase-1 in active SLE patients, we used a fluorescence-labeled inhibitor probe (FLICA), which binds to intracellular active caspase-1 specifically. The medium fluorescence intensity (MFI) of active capase-1 in monocytes of active SLE patients was significantly higher than that of the healthy controls (Fig. 1a, b). Since the production of IL-1β and IL-17A is increased following NLRP3 activation, we then measured serum concentration of IL-1β and IL-17A in these patients. Serum levels of IL-1β and IL-17A in SLE patients were significantly higher than those of healthy controls (Fig. 1c, d). Serum level of IL-1β was correlated with capase-1 activities in active SLE patients (Fig. 1e). Interestingly, the MFI of active capase-1 was also correlated with serum anti-dsDNA antibody level (Fig. 1f), suggesting the possibility of anti-dsDNA antibodies in triggering NLRP3 inflamasome activation. The MFI of caspase-1 was also correlated with disease activity index, the SLEDAI (Fig. 1g).

### Anti-dsDNA antibodies activate NLRP3 inflammasome in monocytes/mocrophages from SLE patients

**Fig. 2:**
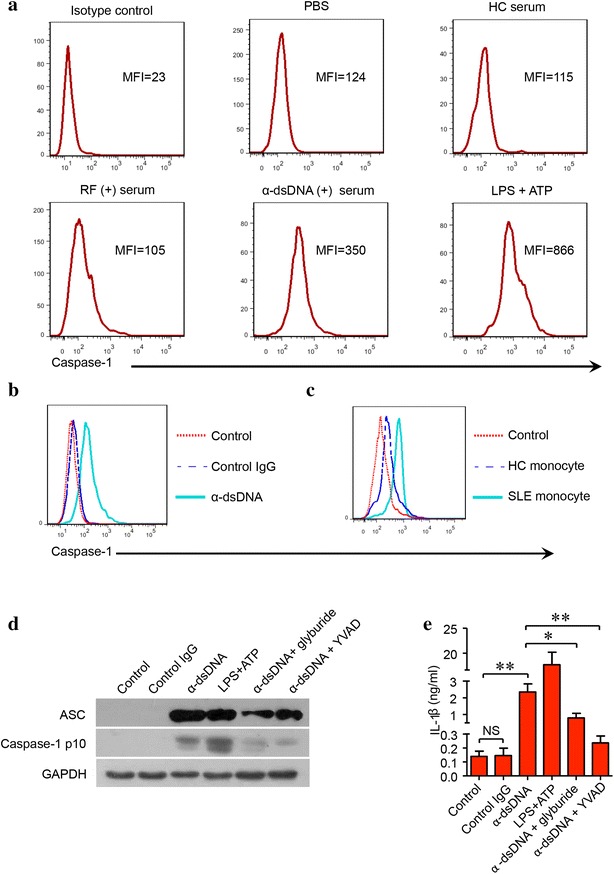
Anti-dsDNA antibodies from SLE patients activated inflammasome in monocytes/macrophages. **a** Monocytes from SLE patients were stimulated with different stimuli (health control serum, RF positive serum, anti-dsDNA antibody-positive serum, LPS + ATP or vehicle control). The activation of caspase-1 was measured by flow cytometry. Anti-dsDNA antibody-positive serum from SLE patients increased the activity of caspase-1 in monocytes. Healthy control serum or RF-positive serum from RA patients did not affect the activity of caspase-1. **b** SLE monocytes were stimulated with IgG from healthy controls or anti-dsDNA antibodies from SLE patients. Anti-dsDNA antibodies increased the activity of caspase-1. Healthy control IgG had no effect on the activity of caspase-1 in monocyte. **c** Monocytes from healthy controls or SLE patients were stimulated with anti-dsDNA antibodies. Activation of caspase-1 in SLE monocytes was significantly increased as compared to monocytes from healthy controls. **d**, **e** SLE Monocyte derived macrophages were stimulated with anti-dsDNA antibodies. Anti-dsDNA antibodies stimulated the activation of inflammome, which can be inhibited by NLRP3 inhibitor glyburide or caspase-1 inhibitor Ac-YVAD-CMK. Each experiment was repeated for three times. *p < 0.01, **p < 0.001. *α*-*dsDNA* anti-dsDNA antibodies; *RF* rheumatoid factor

Monocytes isolated from SLE patients were stimulated with different stimuli (serum from healthy controls, RF-positive serum from RA patients, anti-dsDNA antibody-positive serum from SLE patients and LPS + ATP). Anti-dsDNA antibody-positive serum stimulation resulted in the activation of inflammasome in monocytes. However, healthy control serum or RF-positive serum did not activate inflammasome as measured by flow cytometry by using FLICA (Fig. 2a). Previous study showed that anti-dsDNA antibodies from SLE patients stimulated the overproduction of IL-1 from mononuclear cells [17]. To study the mechanism of anti-dsDNA antibodies in the production of IL-1, anti-dsDNA antibodies isolated from active SLE patients were used to stimulate monocytes. There was marked activation of inflammasome in monocytes stimulated with anti-dsDNA antibodies as measured by FLICA (Fig. 2b). On the other hand, anti-dsDNA antibodies also activated inflammasome in monocytes from healthy controls (Fig. 2c). Monocytes from active SLE patients had higher activation level of NLRP3 inflammasome following anti-dsDNA antibody stimulation than that from healthy controls (Fig. 2c). Furthermore, anti-dsDNA antibodies stimulated the activation of NLRP3 inflammasome in monocyte-derived macrophages (Fig. 2d), resulting in the production of IL-1β (Fig. 2e). Glyburide, which specifically inhibits NLPR3 inflammasome activation [10], was used to inhibit NLRP3, and capase-1 inhibitor YVAD was used to inhibit caspase-1. Glyburide and YVAD significantly inhibited the activation of NLRP3 inflammasome and the production of IL-1β stimulated by anti-dsDNA antibodies (Fig. 2d, e). It implied that anti-dsDNA antibodies activated NLRP3 inflammasome in monocytes/macrophages from SLE patients.

### Anti-dsDNA antibodies activate NLPR3 inflammasome in macrophages via production of mitochondria-derived ROS

**Fig. 3:**
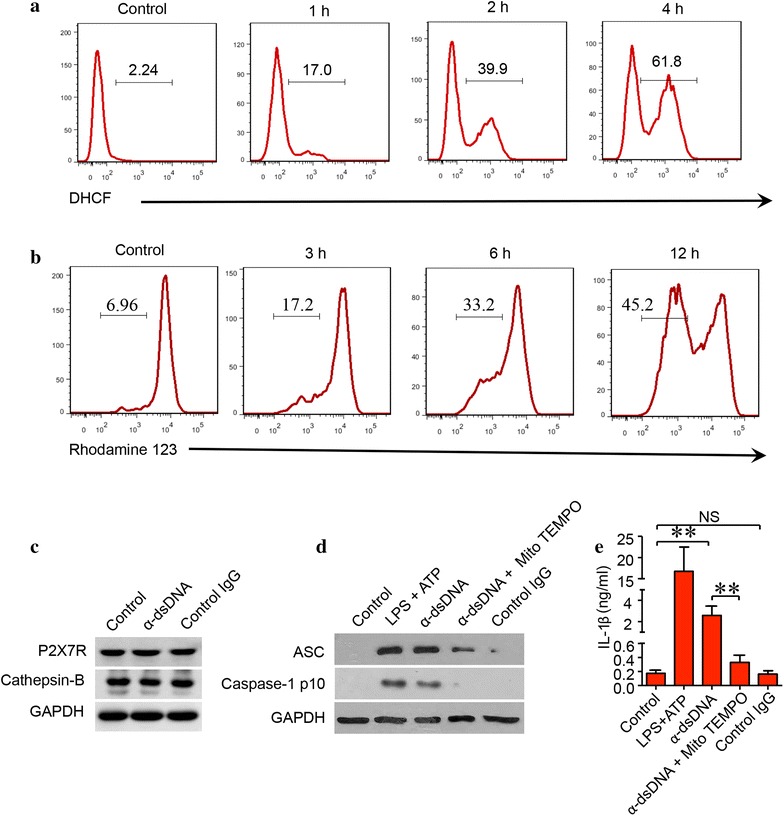
Anti-dsDNA antibodies activated inflammasome by increasing the production of ROS. **a** Monocyte-derived macrophages from SLE patients were stimulated with anti-dsDNA antibodies for different duration and incubated with DHCF-DA. Anti-dsDNA antibodies increased the production of ROS in macrophages. **b** Monocyte-derived macrophages from SLE patients were stimulated with anti-dsDNA antibodies and incubated with Rhodamine 123. Anti-dsDNA antibodies decreased mitochondria membrane potential (ΔΨm) in macrophages. **c** Monocyte-derived macrophages from SLE patients were stimulated with anti-dsDNA antibodies and the expression of P2X7R and cathepsin-K was measured by western blot. There was no significant change in the expression of P2X7R and cathepsin-K when stimulated with anti-dsDNA antibodies. **d**, **e** The activation of inflammasome in macrophages was inhibited by mitochondrial-targeting Mito-TEMPO when stimulated with anti-dsDNA antibodies. Each experiment was repeated for three times. **p < 0.001. *α*-*dsDNA* anti-dsDNA antibodies

Monocyte-derived macrophages from SLE patients were stimulated with anti-dsDNA antibodies for different duration. Macrophages were incubated with DCHF-DA and analyzed by flow cytometry. The production of ROS started to increase 1 h after anti-dsDNA antibody stimulation and continued to increase up to 4 h (Fig. 3a). In addition, anti-dsDNA antibodies from active SLE patients affected the function of mitochondria as evident by decreased mitochondria membrane potential (ΔΨm) (Fig. 3b). However, there was no increase in the expression of P2X7R and cathepsin-B, the upstream molecules of NLRP3 inflammasome (Fig. 3c). The activation of NLRP3 inflammasome stimulated by anti-dsDNA antibodies was inhibited by mitochondria-targeting antioxidant Mito-TEMPO. The expression of ASC and caspase-1 p10 and the production of IL-1β were significantly reduced by pre-treatment with Mito-TEMPO in the culture system (Fig. 3d, e).

### Anti-dsDNA antibodies regulated the activation of NLRP3 inflammasome by binding to TLR4

**Fig. 4:**
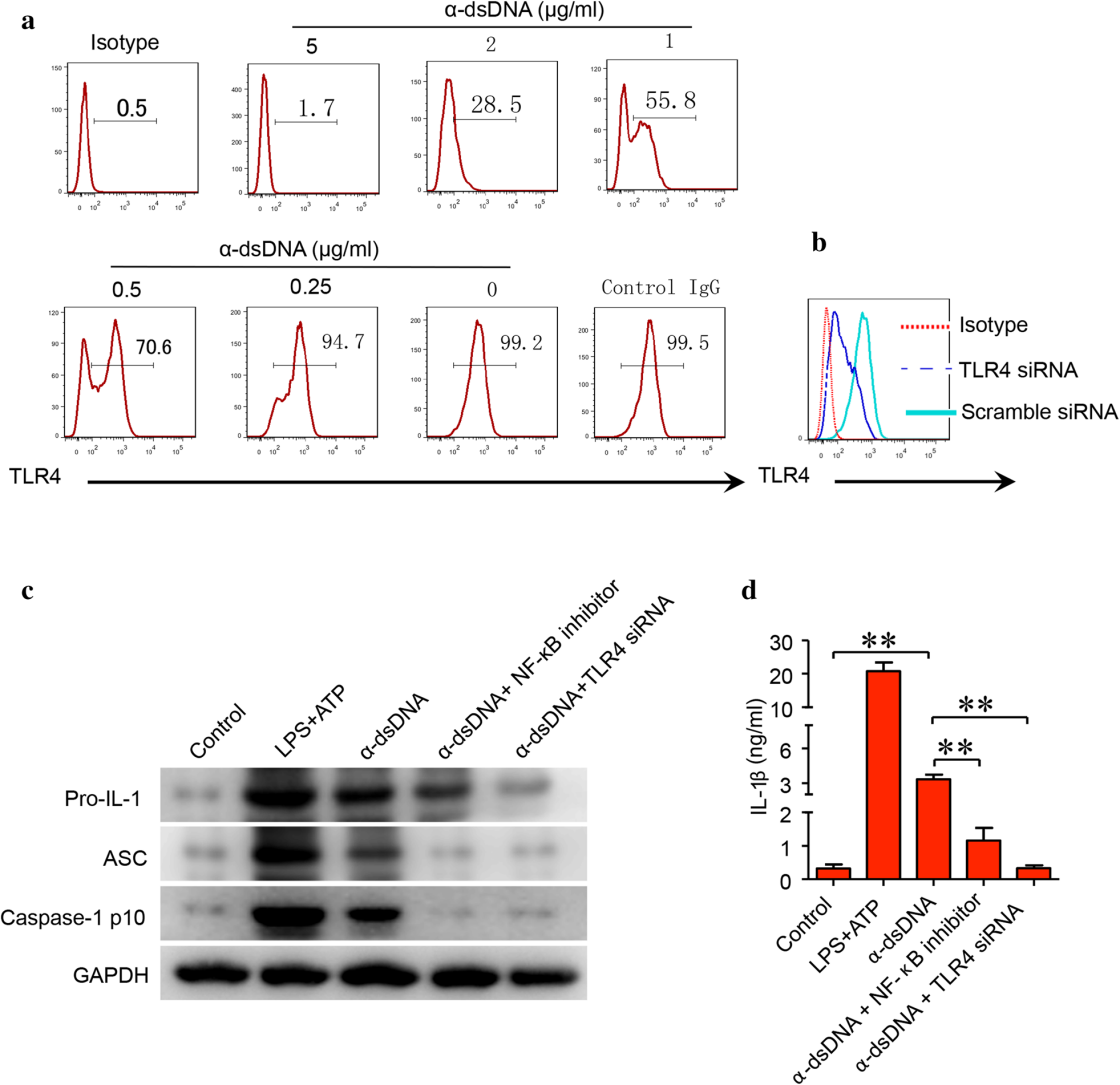
TLR4-NF-κB signal pathway regulated the activation of inflammasome stimulated by anti-dsDNA antibodies. **a** SLE monocyte-derived macrophages were incubated with anti-CD16/CD32/CD64 antibodies to block FcR before incubated with different concentrations of anti-dsDNA antibodies. Cells were then stained with TLR4 antibody. Data was acquired by flow cytometry. Anti-dsDNA antibodies bound to TLR4 as shown by the flow charts. **b** THP-1 cells were treated with TLR4-specific siRNA or scramble siRNA. The depletion of TLR4 was confirmed by flow cytometry. **c**, **d** THP-1 cells were stimulated with anti-dsDNA antibodies and the activation of inflammasome was measured by western blot. Anti-dsDNA antibodies stimulated the activation of inflammasome in THP-1 cells, which was inhibited by depleting TLR4 or by inhibition of NF-κB. Each experiment was repeated for three times. **p < 0.001. *α*-*dsDNA* anti-dsDNA antibodies

Monocytes/macrophages are rich in Fc receptor (FcR). IgG can bind to cell surface by interacting with FcR through Fc region. To rule out the binding of IgG to FcR, FcR was blocked by using anti-CD16/CD32/CD64 antibodies before incubation with anti-TLR4 antibody. Cells were then pre-treated with anti-dsDNA antibodies before incubation with anti-TLR4 antibody. The binding of anti-TLR4 antibody to monocyte-derived macrophages was completely blocked by pre-treatment with anti-dsDNA antibodies but not with control IgG (Fig. 4a). The activation of NLRP3 inflammasome stimulated by anti-dsDNA antibodies was further confirmed in THP-1 cell line. TLR4-specific siRNA was used to deplete TLR4 in THP-1 cells and gene silencing was confirmed by flow cytometry (Fig. 4b). Anti-dsDNA antibodies stimulated the activation of NLRP3 inflammasome as evident by increased expression of Caspase-1 p10 in THP-1 cell line (Fig. 4c). The expression of caspase-1 p10 was significantly inhibited by TLR4-specific siRNA (Fig. 4c). The production of IL-1β was also significantly decreased (Fig. 4d). Interestingly, the expression of pro-IL-1 and ASC was also significantly inhibited by TLR4-specific siRNA. Furthermore, IκB kinase inhibitor peptide significantly decreased the expression of pro-IL-1 and the activation of NLRP3 inflammasome (Fig. 4c, d). These data implied that anti-dsDNA antibodies activated NLRP3 inflammasome by binding to TLR4 in macrophages.

### Anti-dsDNA antibodies activated NLRP3 inflammasome in vivo and impaired the balance of Th17/Treg cells

**Fig. 5:**
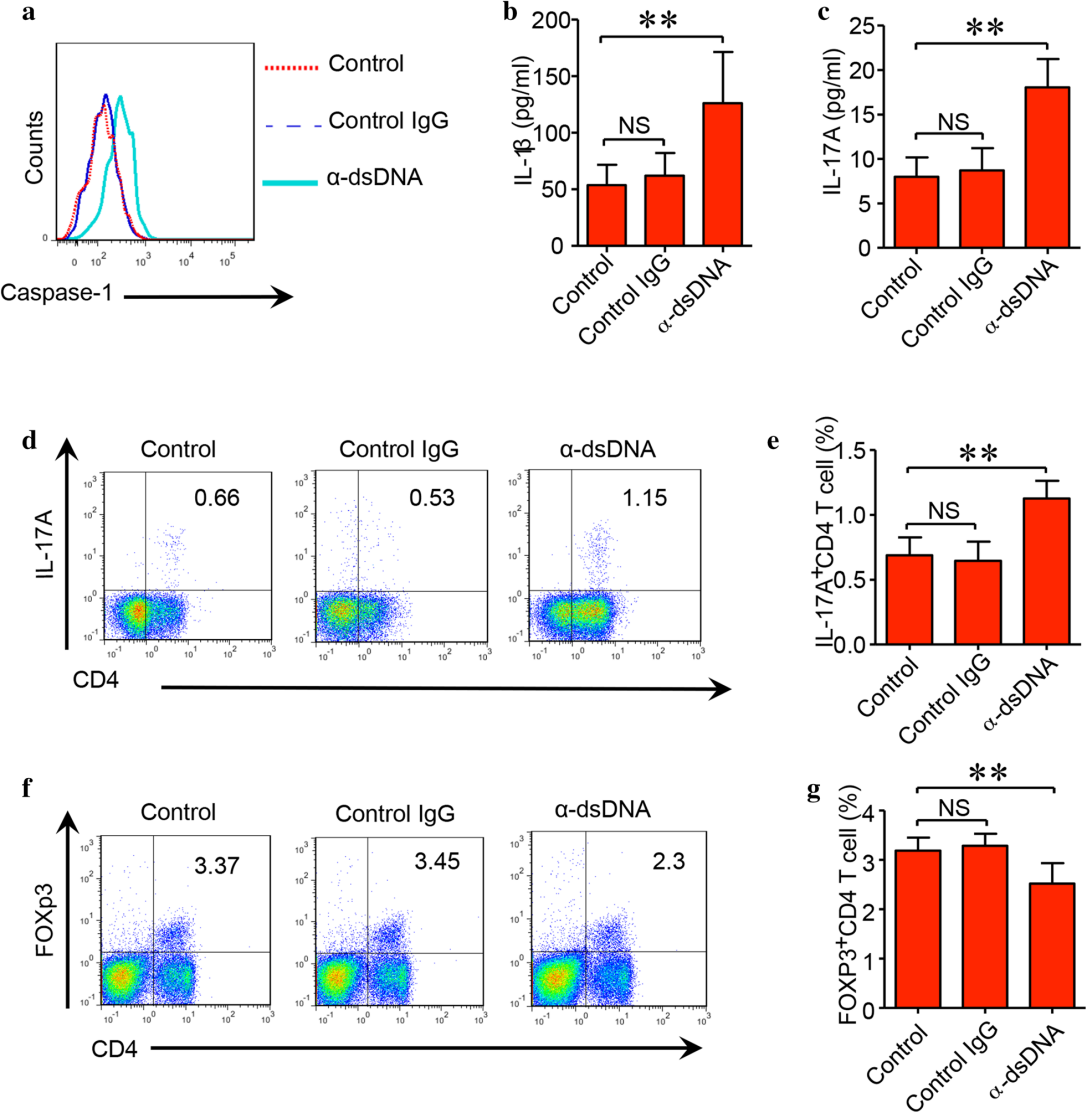
Anti-dsDNA antibodies stimulated the activation of inflammasome in vivo and impaired the balance of Th17/Treg. Anti-dsDNA antibodies isolated from active SLE patients, vehicle or control IgG from healthy patients were injected into female (NZB × NZW) F1 mice (n = 8) twice. Spleens and blood were collected 7 days later. **a** The activation of inflammasome in monocytes was measured by flow cytometry. Cells were gated on CD11b^+^ cells. Anti-dsDNA antibodies activated the inflammasome in vivo as compared to control IgG or vehicle. **b** The concentration of IL-1β in the serum was significantly higher in the serum of anti-dsDNA antibody-injected mice compared to control IgG or vehicle-injected mice. **c** Serum concentration of IL-17A was also significantly higher in anti-dsDNA antibody-injected mice. **d**, **e** The frequency of Th17 cells was significantly higher in anti-dsDNA antibody-injected mice as compared to control IgG or vehicle-injected mice. **f**, **g** The frequency of CD4^+^Foxp3^+^ Treg cells was significantly lower in anti-dsDNA antibody-injected mice as compared to control IgG or vehicle-injected mice. **p < 0.001. *α*-*dsDNA* anti-dsDNA antibodies

Anti-dsDNA antibodies were injected into female (NZB × NZW) F1 mice. Healthy control IgG or vehicle was used as controls. Anti-dsDNA antibodies stimulated the activation of inflammasome in monocytes of mice receiving anti-dsDNA antibody injection (Fig. 5a). In the anti-dsDNA antibody-injected mice, serum concentration of IL-1β and IL-17A was significantly higher as compared to control IgG or vehicle injected mice (Fig. 5b, c). The frequency of Th17 cells significantly increased in anti-dsDNA antibody-injected mice (Fig. 5d, e). However, the frequency of CD4^+^Foxp3^+^ Treg cells significantly decreased in anti-dsDNA antibody-injected mice compared to control IgG or vehicle-injected mice (Fig. 5f, g).

## Discussion

The activation of NLRP3 inflammasome is associated with the pathogenesis of autoimmune diseases [29]. It has been shown that NLRP3 inflammasome activation is involved in the pathogenesis of lupus mouse models [19] and in human [30]. NLRP3 inflammasome can be activated by diverse exogenous stimuli, such as silica [31]; or endogenous stimuli, such as islet amyloid polypeptide [10]. In the present study, we found that NLRP3 inflammasome was activated in monocytes from SLE patients. In addition, anti-dsDNA antibodies isolated from active SLE patients stimulated caspase-1 activation and increased IL-1β production in monocytes/macrophages, suggesting that anti-dsDNA antibodies activate NLRP3 inflammasome. Our finding are in consistency with the report by Shin et al. [18] showing that dsDNA alone could not induce IL-1β production and could induce IL-1β production only in the presence of serum containing anti-dsDNA antibodies. Interestingly, we found that the activation level of NLRP3 inflammasome in monocytes was correlated with serum anti-dsDNA antibody level and disease activity in SLE patients.

Anti-dsDNA antibodies were present in the blood before disease onset and the level of anti-dsDNA antibodies is correlated with disease activity [15]. Injection of anti-dsDNA antibodies from SLE patients promoted disease progression in NZBW F1/J mice [16]. To further confirm the pathogenic relevance of our in vitro findings and those from Shin MS et al. [18], we injected anti-dsDNA antibodies from SLE patients into female (NZB × NZW) F1 mice. This resulted in the in vivo activation of NLRP3 inflammasome in monocytes and increased production of IL-1β. Injection of allogenic IgG into mice might result in non-specific activation of NLRP3 inflammasome. However, this is unlikely since injection of human IgG from healthy controls did not stimulate the activation of NLRP3 inflammasome or increase the production of IL-1β. Therefore, these data suggest that anti-dsDNA antibodies may be involved in the pathogenesis of lupus by activating NLRP3 inflammasome.

The activation of NLRP3 inflammasome has been associated with different mechanisms: production of ROS [32], potassium efflux [33] and the release of cathepsin B into cytoplasm [34]. The production of ROS had a critical role for the development of renal injuries in SLE patients [35, 36]. However, what cause the production of ROS and how ROS promotes the disease is still not clear. In this study, anti-dsDNA antibodies were showed to stimulate the production of ROS in monocytes/macrophages. In addition, the activation of NLRP3 inflammasome in monocytes/macrophages was inhibited by mitochondria-targeting antioxidant Mito-TEMPO, suggesting that the mitochondrial ROS production stimulated by anti-dsDNA antibodies mediates the activation of NLRP3 inflammasome. However, we did not observe increased expression of K^+^ channel P2X7R or cathepsin B in monocytes/macrophages stimulated by anti-dsDNA antibodies.

Mitochondria is the major organelle that generates ROS and decreased mitochondria membrane potential is related to ROS production [37]. It has been demonstrated that mitochondria function was in associate with the activation of NLRP3 inflammasome [38] and mitochondria-derived ROS is responsible for the activation of NLRP3 inflammasome [39]. In this study, we found the anti-dsDNA antibody stimulation resulted in decreased mitochondria membrane potential, suggesting that activation of NLRP3 inflammasome by anti-dsDNA antibodies was mediated through stimulating ROS production in mitochondria.

Recent data showed that NF-κB signal priming is essential for the activation of NLRP3 inflammasome [40]. The stimulation of TLR4 leads to the activation of NF-κB and expression of IL-1 [41]. Previous study showed that TLR9 and NF-κB were essential in producing IL-β by human monocytes in response to self dsDNA and anti-dsDNA antibodies [18]. In this study, anti-dsDNA antibodies were shown to bind to TLR4, leading to increased expression of pro-IL-1 and the activation of NLRP3 inflammasome, and finally the secretion of IL-1β from monocytes/macrophages. Gene silencing of TLR4 or inhibition of NF-κB signal significantly reduced the expression of pro-IL-1 and ASC, and suppressed the formation of active caspase-1 in THP-1 cell-derived macrophages. These results indicate that anti-dsDNA antibodies bind to TLR4, activate TLR4-NF-κB and NLRP3 inflammasome signal pathway.

Th17 is a newly identified subpopulation of CD4 T cells that is expanded in SLE patients and plays an important role in the pathogenesis of SLE [42]. Previous study showed that the activation of NLRP3 inflammasome promoted the differentiation of Th17 cells [26]. In this study, the serum level of IL-17A was significantly higher in active SLE patients. The activation of NLRP3 inflammasome might be involved in the differentiation of Th17 cells. This speculation is supported by the in vivo data. Injection of anti-dsDNA antibodies into female (NZB × NZW) F1 mice resulted in NLRP3 activation, increased production of IL-1β and IL-17A and increased Th17/Treg ratio. The activation of NLRP3 inflammasome stimulated by anti-dsDNA antibodies might promote disease severity by expanding Th17 cells and screwed the balance of Th17/Treg.

## Conclusion

Anti-dsDNA antibodies from SLE patients can activate NLRP3 inflammasome in monocytes/macrophages by binding to TLR4 and activating TLR4-NF-κB signal pathway. The activation of NLRP3 inflammasome stimulated by anti-dsDNA antibodies is dependent on the production of mitochondrial ROS. This study provides new evidence in the pathogenesis of SLE that might be used as a potential treatment target for the disease.

## Abbreviations

SLE: systemic lupus erythematosus
RF: rheumatoid factor
ASC: apoptosis-associated speck like protein
Anti**-**dsDNA: anti-double stranded DNA
SLEDAI: systemic lupus erythematosus disease activity Index
PBMC: peripheral blood mononuclear cells
RA: rheumatoid arthritis
TLR4: toll like receptor 4
ROS: reactive oxygen species
MFI: medium fluorescence intensity
